# Diversity and Host Specificity Revealed by Biological Characterization and Whole Genome Sequencing of Bacteriophages Infecting *Salmonella enterica*

**DOI:** 10.3390/v11090854

**Published:** 2019-09-14

**Authors:** Karen Fong, Denise M. Tremblay, Pascal Delaquis, Lawrence Goodridge, Roger C. Levesque, Sylvain Moineau, Curtis A. Suttle, Siyun Wang

**Affiliations:** 1 Food, Nutrition and Health, The University of British Columbia, Vancouver, BC V6T 1Z4, Canada; karen.fong@ubc.ca; 2 Félix d’Hérelle Reference Center for Bacterial Viruses, Faculté de Médecine Dentaire, Université Laval, Québec City, QC G1V 0A6, Canadasylvain.moineau@bcm.ulaval.ca (S.M.); 3 Groupe de Recherche en Écologie Buccale, Faculté de Médecine Dentaire, Université Laval, Québec City, QC G1V 0A6, Canada; 4 Agriculture and Agri-Food Canada, Summerland, BC V0H 1Z0, Canada; pascal.delaquis@canada.ca; 5 Food Science Department, University of Guelph, Guelph, ON N1G 2W1, Canada; lawrence.goodridge@mcgill.ca; 6 Institut de Biologie Intégrative et des Systèmes (IBIS), Université Laval, Quebec City, QC G1V 0A6, Canada; rclevesq@ibis.ulaval.ca; 7 Département de Biochimie, de Microbiologie, et de Bio-Informatique, Faculté des Sciences et de Génie, Université Laval, Québec City, QC G1V 0A6, Canada; 8 Departments of Earth, Ocean and Atmospheric Sciences, Microbiology and Immunology, and Botany, and the Institute for Oceans and Fisheries, The University of British Columbia, Vancouver, BC V6T 1Z4, Canada

**Keywords:** Bacteriophage, *Salmonella*, biocontrol, comparative genomics, phage diversity

## Abstract

Phages infecting members of the opportunistic human pathogen, *Salmonella enterica*, are widespread in natural environments and offer a potential source of agents that could be used for controlling populations of this bacterium; yet, relatively little is known about these phages. Here we describe the isolation and characterization of 45 phages of *Salmonella enterica* from disparate geographic locations within British Columbia, Canada. Host-range profiling revealed host-specific patterns of susceptibility and resistance, with several phages identified that have a broad-host range (i.e., able to lyse >40% of bacterial hosts tested). One phage in particular, SE13, is able to lyse 51 out of the 61 *Salmonella* strains tested. Comparative genomic analyses also revealed an abundance of sequence diversity in the sequenced phages. Alignment of the genomes grouped the phages into 12 clusters with three singletons. Phages within certain clusters exhibited extraordinarily high genome homology (>98% nucleotide identity), yet between clusters, genomes exhibited a span of diversity (<50% nucleotide identity). Alignment of the major capsid protein also supported the clustering pattern observed with alignment of the whole genomes. We further observed associations between genomic relatedness and the site of isolation, as well as genetic elements related to DNA metabolism and host virulence. Our data support the knowledge framework for phage diversity and phage–host interactions that are required for developing phage-based applications for various sectors, including biocontrol, detection and typing.

## 1. Introduction

Bacteriophages are the most abundant biological entity on Earth and have been estimated to kill 20% to 25% of microbes daily [1,2,3,4]. Moreover, phages are key contributors to bacterial ecology and evolution through obligate parasitism, using either lytic or temperate life cycles thereby resulting in direct or delayed lysis of bacterial hosts, respectively [5]. Phage–host interactions have contributed vastly to genetic flux through horizontal gene transfer that is responsible for the dissemination and acquisition of important bacterial phenotypes, such as enhanced colonization of the human gut epithelium, antimicrobial resistance and toxin production [2,6].

Phage diversity is immense and the global phage gene pool likely represents the greatest biodiversity and largest potential source of novel genes, providing new insights on phage diversity and evolutionary relationships in disparate environments [2,7,8]. Moreover, this vast diversity is a potential reservoir of antibacterial agents for developing “phage therapies” or “biocontrol” strategies to control bacterial pathogens. Phage-based biocontrol of bacterial pathogens in foods and food processing environments is an attractive alternative to using synthetic antimicrobial agents or physical disinfection treatments that can have harmful effects on humans, animals and plants [9,10,11]. At least, phages most suited to this purpose should exhibit a broad host range and are free of genes encoding for lysogeny and resistance to antimicrobial agents and/or virulence [10].

Non-typhoidal *Salmonella enterica* is a foodborne pathogen causing high rates of mortality and morbidity worldwide [12,13]. Globally, bacteria in the genus *Salmonella* cause 93 million enteric infections and 155,000 diarrheal deaths each year [12], and although there are animal reservoirs including poultry and swine [8], its presence in other food products such as nuts, produce and ready-to-eat products [14] confirms that it can adapt to diverse environments [15].

Comparative genomics approaches have been used to aid in the development of phage-based products targeting several genera including *Acinetobacter*, *Pseudomonas*, *Mycobacterium*, *Lactococcus*, *Vibrio,* and *Salmonella* [2]. These analyses provided insights at genomic and phylogenetic levels (e.g., phage relatedness and the elucidation of novel genetic elements), associations among phage communities across disparate environments, and elucidation of novel phage–host interactions [2,16,17,18,19,20]. Nevertheless, an in-depth understanding of *Salmonella* phage diversity and phenotype-genotype characteristics is lacking. Here, we present comparative phenotypic, genomic and phylogenetic analyses of 45 new phage isolates from British Columbia, Canada, that infect non-typhoidal strains of *Salmonella*.

## 2. Materials and Methods 

### 2.1. Bacterial Strains and Growth Conditions

*Salmonella* strains (*n* = 61) were obtained from various sources, including the International Life Sciences Institute, the *Salmonella* foodborne syst-OMICS database, or were isolated from the Lower Mainland of British Columbia (Table S1). Strains were maintained at −80 °C in Brain–Heart-Infusion broth (BD/Difco, East Rutherford, NJ, United States) supplemented with 20% glycerol. Working stocks were prepared and maintained on tryptic soy agar (TSA; BD/Difco) at 4 °C for a maximum of one month. Fresh overnight liquid cultures were prepared prior to each experiment by inoculating an isolated colony into 10 mL tryptic soy broth (TSB; BD/Difco). Cultures were incubated for 16 h at 37 °C with shaking at 170 rpm.

### 2.2. Bacteriophage Isolation and Purification

**Figure 1:**
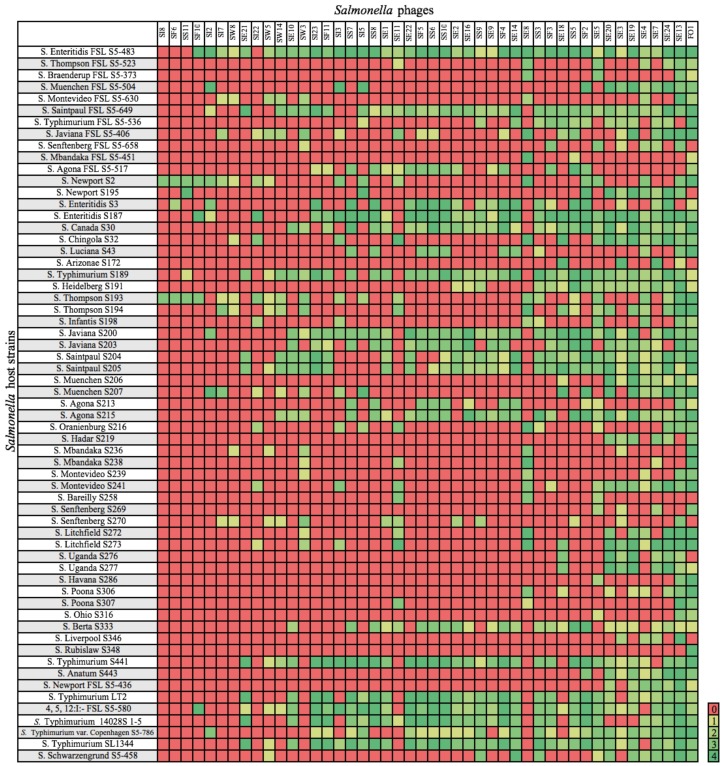
Host range of isolated phages. *Salmonella* strains susceptible to phage infection are indicated by a clearing of 1 to 4; 0 = no lysis.

Bacteriophages were isolated from sediment (S), cattle feces (F), sewage effluent (E), irrigation water (I), and water tanks from an aquaculture facility (W) in British Columbia, Canada, and as specified in the phage name (Figure 1 and Figure S1). Four broad-host range phages SI1, SF1, SS1, and SS4 were isolated previously [21]. 

Sample enrichment, phage isolation and purification were carried out as described elsewhere [21]. Briefly, an effort was made to enrich the abundance of phage by mixing 10 g of sample, 90 mL TSB and 1 mL of a cocktail of 7 indicator strains of *Salmonella* (Table S1) that were grown for 16 h as described above. Serotypes represented by these strains include high-risk types important to food safety and have exhibited high prevalence in human cases of salmonellosis [22,23]. Suspensions were then incubated at 37 °C for 22 ± 2 h. The phage-enriched samples were spun at 4000× *g* to remove bacteria, and the supernatant passed through a 0.45-µm pore-size polyethersulfone filter membrane (Pall Corporation, Port Washington, NY, United States). Afterwards, 100 µL of filtrate was mixed with 300 µL of each of the indicator *Salmonella* strains (diluted 10-fold after growing for 16 h) and 4 mL of 0.7% TSA top agar, according to the double-agar overlay method [24]. Plates were then incubated at 37 °C for 18 ± 2 h for plaque visualization. Plaques were lifted from the agar surface using a truncated sterile pipette tip and re-suspended in 200 µL salt–magnesium (SM) buffer (0.05 M Tris-HCl; 0.1 M NaCl and 0.01 M MgSO_4_; adjusted to pH 7.5). Suspensions were allowed to rest for at least 6 h at room temperature. Double agar overlays were then prepared with the suspension as described previously [21]. A minimum of 3 single plaque isolations was performed in series to obtain a clonal phage isolate. Phages were subsequently concentrated by centrifugation and stored at 4 °C until further analyses.

### 2.3. Host Range Determination

Purified phage lysates were standardized to a concentration of 10^9^ PFU/mL in SM buffer as recommended previously [24]. Felix-O1, a strictly lytic phage, was used as a control as it infects most members of the Salmonellae [25]. Host range was determined by spotting 5 µL of lysate, in duplicate, on a lawn of *Salmonella* cells (representing rare and common strains and serotypes) [26] grown for 16 h at 37 °C (*n* = 61 strains; Figure 1). The drops were allowed to dry at room temperature prior to incubation at 37 °C for 18 ± 2 h. Zones of cell lysis were assessed with a scaling system [21,22,27], where 0 indicated a zone with complete turbidity (no infection) and +4 indicated a completely clear zone with no turbidity. These values were converted into a heat map as shown in Figure 1. 

### 2.4. Phage DNA isolation

Prior to DNA isolation, 1 mL of the lysates was filtered with a 0.45-µm pore-size cellulose–acetate membrane (Pall Corporation). Subsequently, 5 µL of both 10X RNAse A (Invitrogen, Carlsbad, CA, United States) and 1X DNAse (Invitrogen) were added to the filtered lysates for removal of contaminating host nucleic acid. MgSO_4_ was also added to a final concentration of 10 mM and the suspension was incubated at 37 °C for 30 min. Then, 100 µL of lysis solution (2.5% sodium dodecyl–sulfate, 0.25 M EDTA and 0.50 M Tris-HCl (pH 9.0)) was added to the mixture, followed by incubation at 65 °C for 30 min. Subsequently, 125 µL of 8 M potassium acetate (Amresco, Solon, OH, United States) was added and the suspensions were placed on ice for 30 min, then spun at 17,000 rpm (27,141× *g*) for ten min at 4 °C. Afterwards, 500 µL of phenol–chloroform (Amresco) was added, the contents were mixed on a vortex for 1 min, and spun at 14,000 rpm (18,407× *g*) for 10 min at room temperature. The upper phase containing the DNA was carefully transferred to a clean microcentrifuge tube and an equal volume of isopropanol (Amresco) was added, followed by centrifugation at 17,000 rpm (27,141× *g*) for 10 min at 4 °C. The supernatant was discarded, and the pellet washed 3 times with 70% ethanol and allowed to dry for 15 min. Finally, the pellet was re-suspended in 20 µL Tris-HCl (pH 8.0) and stored at −80 °C until analyzed [28].

### 2.5. DNA Sequencing and Annotation Workflow

The DNA library was prepared using the Nextera XT DNA Library Preparation Kit (Illumina, Hayward, CA, United States) according to the manufacturer’s instructions and shotgun-sequenced using the Illumina MiSeq platform with the MiSeq Reagent Kit v2 (Illumina). Contigs were assembled de novo from the paired-end reads with the Ray assembler version 2.2.0 [29]. Depth of sequencing in the newly isolated phages ranged from 40- to 2564-fold coverage (Table S2).

Open reading frames (ORFs) were identified and annotated with the Rapid Annotation using Subsystems Technology (RAST) pipeline [30]. Annotations were also subsequently verified using the BLASTp algorithm (NCBI), employing an E-value cut-off of 0.01 [31].

### 2.6. Genomic Analysis

Genomic analysis was conducted on the phage genomes to identify phages with desirable characteristics for biocontrol purposes and also to probe the biodiversity of our novel isolates. An in silico approach was taken to predict phage morphotypes by comparison with closely related phage genomes using the BLASTp algorithm (NCBI). Genomes that were most closely related (i.e., possessing the highest E-value and >50% query coverage) were chosen to aid in assigning newly sequenced phages to putative families. ARAGORN was used to identify genes encoding putative tRNAs, which employs heuristics and homology comparisons with tRNA consensus sequences for prediction of the tRNA secondary structure [32].

Phylogenetic trees were constructed in MEGA X [33]. Nucleotide sequences were aligned using the ClustalW algorithm and the phylogenetic tree constructed using the Maximum-Likelihood method employing 1000 bootstrap replicates. Clusters in the phylogenetic trees were identified using ClusterPicker [34] with an inter-cluster threshold of 50% nucleotide identity, as has been demonstrated for other phage genomes [2]. Amino-acid and nucleotide comparisons were conducted for individual phages within clusters using alignment tools such as MEGA X. Visual representations and comparisons of whole genomes were produced using EasyFig [35].

Phages were annotated using a combination of automatic (i.e., RAST) and manual (NCBI BLASTp) approaches. Phages were classified as putatively temperate when a gene encoding integrase (for integration into the bacterial host chromosome) could be identified; whereas, phages without this gene were classified as putatively lytic [20,35].

## 3. Results

The genus *Salmonella* comprises a diverse group of microorganisms with well-characterized pan genomes, routes of transmission, pathogenesis, and epidemiology [20,36]. Despite their potential relevance to the genomic and biological attributes of the genus, there is comparatively little genomic information on *Salmonella* phages [20]. 

### 3.1. Host Range of Phages Infecting Salmonella

Given the diversity of strains in the Salmonellae in terms of disease attribution and pathogenicity, we chose strains representing serotypes that (i) cause the most illnesses (e.g., Enteritidis, Typhimurium); (ii) are rarer (compared to Enteritidis and Typhimurium), yet emerging in North America as subtypes associated with foodborne disease (e.g., Heidelberg, Saintpaul); and (iii) demonstrate multi-drug resistance (e.g., Typhimurium SL1344, Schwarzengrund S5-458) [13,21,26].

Overall, the *Salmonella* strains representing serotypes Enteritidis and Typhimurium were lysed by most of the phages (Figure 1), with S. Enteritidis FSL S5-483 exhibiting the greatest phage susceptibility. All of the antibiotic-resistant strains were susceptible to at least one phage isolate. Moreover, the phages infected some rarer, yet emerging strains [21,26], although a subset of bacterial strains (both within and between serotypes) were resistance to phage infection (Figure 1). 

The phages exhibited a variety of host ranges. Some isolates (e.g., SE21, SE10, SI23) had relatively narrow host ranges, infecting 10, 15 and 16 *Salmonella* strains, respectively; others (e.g., SE13, SE7, SE20) were broader and infected 51, 38, and 35 strains, respectively. Felix-O1 had the broadest host range, infecting 54 of 61 tested strains. Originally isolated in England [37], Felix-O1 is a virulent phage that infects 98.2% of all *Salmonella* strains and is commonly used in diagnostics and typing [38,39]. 

Of the newly isolated phages, SE13, from sewage, had the broadest host range, infecting 51 of 61 strains, including some that were weakly resistant (e.g., S. Liverpool S346) or fully resistant (e.g., S. Arizonae S172) to Felix-O1 infection (Figure 1). All *Salmonella* strains were infected by at least one phage except for S. Rubislaw S348 which was only infected by Felix-O1.

### 3.2. General Genomic Characterization 

Genomic characterization was performed to identify phages that possessed particular genomic features that would be pertinent for biocontrol (i.e, rendering them either unable to be used for food biocontrol and/or possessing genes (e.g., tRNA genes, DNA-replication elements) that could potentially constitute an infection/replication advantage in the host) (Table S3). The complete genomes have been deposited into Genbank with accession numbers: MK761195—MK761199, MK770409—MK770415, MK972685—MK972699, MK972700—MK972717 (Table S4). Additionally, we describe here distinct patterns of diversity that were revealed through our genomic analysis.

**Figure 2:**
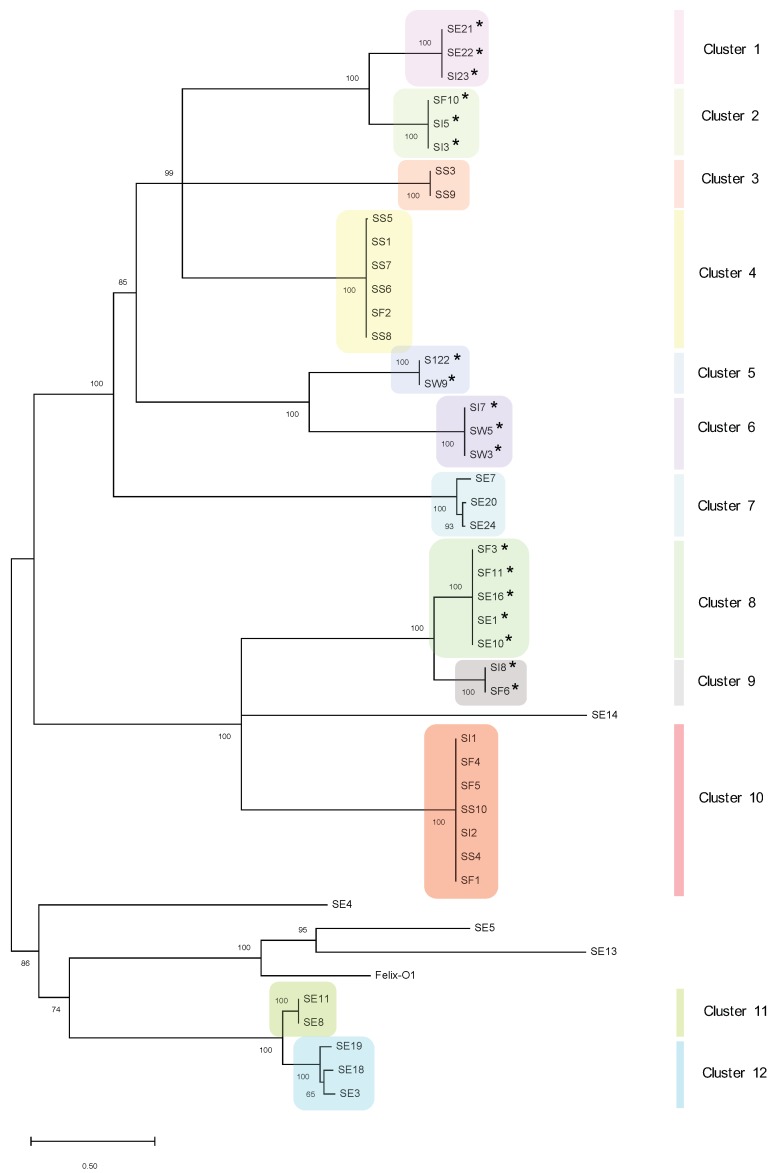
Dendrogram of whole genome nucleotide alignment. Tree was constructed using the ClustalW alignment and the Maximum-Likelihood method in MEGA X with 1000 bootstrap replicates. Bootstrap percentages are shown next to each node. Scale represents the number of nucleotide substitutions per site. Putatively temperate phages are indicated with an asterisk (*).

Phages were organized into 12 distinct clusters on the basis of 50% nucleotide similarity (Figure 2). There were four genomic singletons (including Felix-O1), which did not cluster into any sub-groups, although they exhibited similarity to previously sequenced phages. For instance, singleton phage SE13 showed ~93% nucleotide sequence identity to *Salmonella* phage BP63 (NC_031250), while SE5, interestingly, was 98% similar to Erwinia phage phiEa21-4 (NC_015292) (Table 1). Interestingly, phages of Cluster 6 did not show any significant matches to previously isolated phages, indicating the presence of a novel genus or cluster.

Overall, clusters of phages with a high degree of genetic similarity could be isolated from disparate sampling sites. For instance, phages in Cluster 8 were isolated from sewage effluent and cattle feces, while Cluster 10 contained phages from sediment, irrigation water and cattle feces (Figure 2). However, phages from sewage also occurred across different clusters (e.g., Clusters 1, 7, 8, 11, and 12) or could not be assigned a specific cluster, indicating sewage is a rich source of phage diversity.

A lysogeny module containing a gene encoding for integrase (i.e., responsible for integration into the host chromosome) was identified in 18 of the 45 phages, indicating a possible temperate lifestyle (Figure 2 and Table S3). Furthermore, these phages were also isolated from diverse environments (Table S5). The putative temperate phages belonged to seven clusters: Clusters 1, 2, 5, 6, 8, and 9 (Figure 2). Accessory proteins associated with recombination (e.g., C protein and Cox proteins) were also identified in the lysogeny modules of Cluster 6 phages. 

Interestingly, we also noted similar clustering patterns upon comparison of the whole genome and major capsid protein (MCP) phylogenetic trees (Figure S1). For instance, 5/6 of the phages in Cluster 4 in the whole genome dendrogram (Figure 2) are also clustered together in Cluster 4 of the MCP dendrogram (Figure S1). All phages within Clusters 5 and 6 on both figures are also grouped together similarly. Clusters 8 and 9 of the whole genome dendrogram are also grouped together to form Cluster 7 in the MCP dendrogram. On the basis of the MCP, singletons SE13, SE4, and SE14, as identified in Figure 2, are either grouped together or with other phages (Cluster 4, Figure S1), indicating the conservation of this shared core gene. Given the patterns of clustering, these results suggest the MCP may be suitable as a phylogenetic marker for whole genome clustering. 

### 3.3. Phage Classifications 

The taxonomic assignments of 45 newly isolated *Salmonella* phages were predicted by in silico analysis (Table 2) as has been performed by others [40]. For those phages that could be assigned a taxonomic rank, 46.7% (21/45) were classified in the family Siphoviridae, 28.9% (13/45) and 17.8% (8/45) were assigned to the families Podoviridae and Myoviridae, respectively, and 6.7% (3/45) could not be assigned to a family. Previously, phages SI1, SF1, SS1, and SS4 were classified as Siphoviridae, based on morphology determined by transmission electron microscopy [21]. The predicted morphotypes also correlated with cluster analysis of the whole genome and the MCP. For example, Cluster 3 of Figure S1 solely comprised phages predicted as Myoviruses according to the in silico analysis. Additionally, Cluster 4 contained predicted Siphoviruses (Figure S1). 

### 3.4. Identification of Putative Phage tRNAs 

A range of putative tRNA genes were identified in our phage collection (Table 2), with at least one tRNA identified in 36% (16/45) of the phages. The distribution of tRNA-containing phages varied based on the isolation source, with 59% (10/17) of phages from sewage, 25% (2/8) from cattle feces, 38% (3/8) from irrigation water; 22% (2/9) from sediment; and none (0/3) from aquaculture possessing at least one tRNA-encoding gene. The findings suggest that tRNA genes are not uncommon within *Salmonella* phages and may vary geographically.

### 3.5. Genomic Analysis of SE13

Phage SE13 isolated from sewage had the broadest host range of the 45 isolates, lysing nearly all of the antibiotic-resistant strains tested (except *S.* Agona S5-517) and some rare strains (e.g., *S.* Poona S306, S307; uncommonly seen in outbreaks) that were resistant to infection from other phages in our collection, including Felix-O1 (e.g., S. Arizonae S172) (Figure 1). SE13 also lysed the serotypes responsible for the highest rates of infection worldwide, *S.* Enteritidis and *S.* Typhimurium [39].

**Figure 3:**
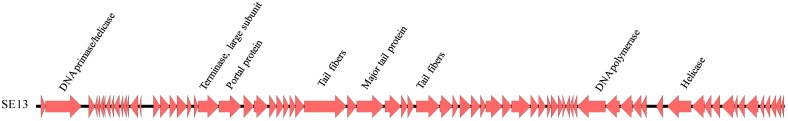
Linear whole-genome representation of phage SE13. Large open reading frames (ORFs) greater than 1000 bp are indicated. Arrows indicate the direction of transcription.

Based on BLASTn, the 52,438 bp genome (G+C = 45.8%) of SE13 revealed 93% identity to *Salmonella* phage BP63, with putative genes involved in structure, host recognition, and metabolism/replication. RAST identified 73 ORFs, suggesting that approximately 9% of the genome is non-coding (Figure 3). RAST assigned functions to 13 of 73 ORFs and subsequent verification with NCBI BLASTp further assigned functions to five additional ORFs, including the major capsid protein. The remaining 55 ORFs were classified as hypothetical (Table S3). No lysogeny-related modules encoding integrase, nor antibiotic-resistance and/or virulence factors were identified.

### 3.6. Genome Size, G+C content and Identification of DNA Metabolism-Related Genes

Cluster analysis separated the phages into 12 groups (Figure 2). ORF prediction using a combination of RAST and BLASTp revealed genetic elements shared among phages in a cluster, as well as distinct genotypes exhibited among clusters. Genome sizes ranged from 30,037 bp to 158,539 bp, and G+C values from 39.2% to 54.4%, in concordance with other phages of *Salmonella* [5,20].

**Figure 4:**
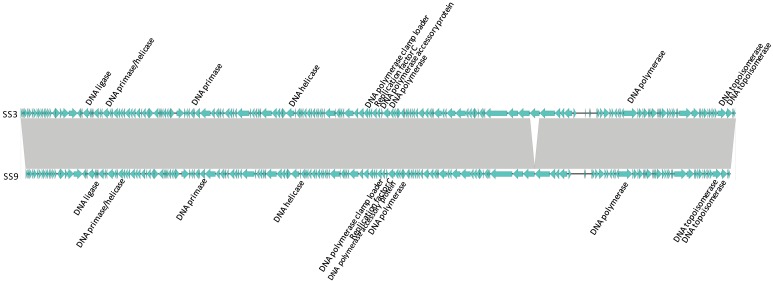
Linear whole genome comparison of phages in Cluster 3. ORFs encoding for DNA replication elements are indicated. Grey regions indicate nucleotide homology of >96%. Directions indicate the direction of transcription.

Phages with genomes >100,000 bp are represented in Clusters 3, 7, 11, and 12; additionally, SE14, a genomic singleton, possesses a genome of 152,926 bp and 198 ORFs. These large genome phages possess accessory genes encoding for proteins involved in phage replication (e.g., DNA polymerase, DNA helicase, DNA primase, replication factor C, sliding clamp loader subunit) that were adjacent (i.e., modular in its arrangement), or separated by a non-related ORF. Further, these genes are positioned on the same strand, implying they are likely to be transcribed together as part of a module [41]. Phages of Cluster 3 are represented in Figure 4. 

These modules were not identified in clusters of phages with smaller genomes (<50,000 bp), nor in genomic singletons SE13 (52,438 bp), SE4 (53, 494 bp), and SE5 (84,567 bp). However, in some of these phages, a small number of replication-related elements were dispersed throughout the genome as well as duplicate copies of select genes encoding for replication-related functions (e.g., DNA ligase, DNA topoisomerase, DNA helicase). Concordantly, duplicate copies of some of these genes were identified in Clusters 3, 7, 11, and 12, representing phages with larger genomes (Figure 4).

### 3.7. Identification of Genes Encoding for Putative Virulence Factors

Putative virulence factors were identified in three phages (Table 2). Three genes putatively encode virulence-related functions, including a polymyxin resistance protein ArnC (311 amino acids). Additionally, a gene encoding for exopolysaccharide production protein ExoZ (380 amino acids) was identified in phages in Clusters 2 and 11 (Table 2). Lastly, a large virulence protein VriC (1613 amino acids) was identified in phages of Cluster 3 (SS3 and SS9), as well as the genomic singleton, SE14. A nucleotide alignment using ClustalW revealed that vriC in SS3 and SS9 was nearly identical with one nucleotide mismatch at position 4374, while vriC in SE14 was slightly more divergent, possessing 96% identity to that of SS3 and SS9. 

## 4. Discussion

Here we describe 45 newly isolated phages that have important phenotypic and genomic properties for biocontrol and their patterns of diversity. Overall, we observed very diverse host ranges within our collection of phages; with phage SE13 possessing the broadest host range. Screening of putative genes revealed the carriage of genes encoding integrase, virulence and antimicrobial resistance in some phages, although SE13 was devoid of these genetic elements. A cluster analysis of the phage genomes revealed an abundance of diversity and also novel associations between genes encoding for replication-related functions, phage genome size, and the G+C content association between phage and *Salmonella* host. Generally, a larger genome size and discordant G+C content was correlated with an increased number of genes encoding for replication-related functions, which may be advantageous if using these phages in biocontrol agents. Lastly, cluster analysis of the major capsid protein revealed a similar grouping pattern with that of the whole genomes, potentiating its use as a marker for genetic relatedness. 

### 4.1. Host Range of Phages Infecting Salmonella

We screened the phage isolates against a panel of 61 *Salmonella* strains to determine their host ranges (Figure 1), as lysis of a broad range of *Salmonella* strains is critical for biocontrol applications [8]. Ideally, phage cocktails are formulated from several phages with broad host ranges [42]. 

Strains representing serotypes Enteritidis and Typhimurium were lysed by most of the phages (Figure 1), which is important considering the global association of these serotypes with salmonellosis [43]. Further, all of the antibiotic-resistant strains were susceptible to at least one phage, which is noteworthy given the need for new strategies to tackle antibiotic resistance. One *Salmonella* strain, *S*. Rubislaw S348 could not be lysed by the newly isolated phages, which may be due to the lack of a suitable receptor, or other phage-resistance mechanisms.

Some groups of phages exhibited unique host ranges. Phages isolated from irrigation water displayed comparatively limited host ranges, which suggests that the available host spectrum is narrower than in other sampled environments. For example, phages from sewage exhibited broader host ranges (Figure 1), especially SE13, which exhibited the broadest host range of the newly isolated phages. This is consistent with sewage containing an abundance of potential hosts and phages [42]. However, phage abundance tends to oscillate with host abundance. The “kill the winner” hypothesis posits that the host that grows most quickly (i.e., the “winner”) will be the most susceptible to phage infection, leading to greater diversity of hosts and phage. Phages with broader host range are better adapted to survive environments where host diversity is high, and the abundance of any given genotype is relatively low, or where environmental conditions and bacterial populations change often, for example sewage [44]. Sewage water and sludge are good sources of phages with broad host ranges, as are some non-sewage sources [42].

It is important to note that the phages isolated here may not represent the proportions of phages in the environment. Laboratory isolation by plaque formation and infection of indicator strains likely sample a subset of phages. However, because the indicator strains used for isolation are common North American foodborne outbreak isolates, the phages described herein may prove highly useful for the control of *Salmonella* in this jurisdiction. 

### 4.2. General Genomic Characterization 

Cluster analysis assigned phages into 12 clusters with four genomic singletons (including Felix-O1) (Figure 2). Overall, some clusters contained phages with high sequence identity that were isolated from disparate sites (e.g., clusters 8 and 10). These observations, coupled with the fact that these phages were isolated from proximate regions within British Columbia, suggest that they may be genetically endemic [20] and/or transmissible via unknown vectors (e.g., wildlife moving among sites). It has also been suggested that phages within a cluster may share common hosts [19], which implies that host populations within a cluster may also be genetically similar [19]; future metagenomic analyses may verify this hypothesis. We also saw evidence of rich diversity across clusters; phages from sewage were assigned to different clusters (e.g., Clusters 1, 7, 8, 11, and 12) or could not be assigned a specific cluster (Figure 2). As wastewater is abundant in nutrients, bacterial hosts, and high rates of horizontal gene transfer, phages isolated from these sites represent a reservoir of novel and diverse genetic materials [45].

A lysogeny module containing a gene encoding integrase was identified in 18 of the 45 phages (Figure 2). Although the use of temperate phages is counter-indicated for some biocontrol applications (e.g., in food systems) [9], phage modification by deletion of lysogeny modules may be considered for other applications, particularly if the phage has a broad host range. It should also be cautioned that in addition to genomic analysis, in vitro transduction assays should be carried out to confirm a virulent lifestyle [20,21].

The MCP was identified and functionally annotated in all phages (Figure S1), suggesting that significant divergence of this protein is constrained due to its important conserved role in capsid assembly [46] and maintenance of the viral capsid structure [38]. Because it represents a core gene and is thus not known to be horizontally transferred, multiple studies have assessed the role of MCP as a phylogenetic marker [16,46,47,48,49], further exaggerated by the fact that there is no benchmark gene used to study phage diversity. Indeed, comparisons of the whole genome and the MCP in our subset of phages show similar clustering patterns (Figure 2 and Figure S1), potentiating its use as an inference gene for genetic relatedness. Furthermore, determination of the MCP sequence may be useful for pre-assignment of novel phages into groups or clusters which share desirable characteristics for biocontrol (e.g., broad host range and infection efficiency) [48,49,50].

### 4.3. Phage Classifications

Electron microscopy and nucleic acid content have largely provided the basis for taxonomic classification [51], with the currently classified viruses exhibiting genomic relatedness in concordance with their morphotypes [52]. This correlation was further corroborated with our cluster analysis (Figure 2). The largest proportion (46.7%) of our phages were assigned to the family *Siphoviridae*, while 28.9% and 17.8% were assigned to the families *Podoviridae* and *Myoviridae*, respectively, and 6.7% could not be assigned to a family (Table 2). The diversity of morphotypes in this collection is important when informing optimal cocktail design. The sensitivity of phage to external factors (e.g., storage, temperature, and pH) varies between morphological families [53], thus cocktails comprising different phage morphotypes should be considered. Additionally, it has been previously reported that *Salmonella* phages of different morphotypes use different host receptors [54], which, when incorporated into a cocktail, diminishes the occurrence of host resistance [55].

### 4.4. Identification of Putative Phage tRNAs 

The presence of tRNA genes is relatively common and has been observed in many phage genomes [56]. It has been proposed that particular tRNA genes benefit phage replication by corresponding to codons used by the phage genome rather than the host [57]. Accordingly, phages with similar codon usages to that of their hosts will not benefit from retention of tRNA genes and would use that of their host [58]. It has also been hypothesized that temperate phages integrate at the position of a host tRNA gene, with the phage tRNA compensating for the interruption in the host tRNA gene [59]. 

At least one tRNA was identified in 36% of the newly isolated phages, consistent with a large-scale comparative analysis of 827 mycobacteriophages, which revealed that 41.4% contained at least one tRNA gene, and that these displayed cluster specificity [56]. In our study, most phages from sewage had at least one tRNA. As sewage represents a rich source of host diversity, having different tRNAs might enhance phage genome replication in multiple hosts [58]. However, different numbers of phages were recovered from each site; thus, these distribution patterns are preliminary.

### 4.5. Genomic Analysis of SE13

Of the newly isolated phages, phage SE13 had the broadest host range (Figure 1) and no evidence of an integrase, and thus was likely lytic. Additionally, as there were no antibiotic-resistance or virulence factors identified, it is a good candidate for biocontrol of *Salmonella*. 

Genetically, SE13 possesses synteny, with predicted ORFs (Table S3) encoding genes for structure (major capsid protein, scaffold protein, tail proteins), packaging (large terminase subunit, portal protein), a lysozyme, and a likely cognate holin in close proximity, as has been identified in other phage genomes [60,61]. Given the broad host range of SE13, the tail fibers are of specific interest. ORFs 31 and 45 encode for putative tail fibers of 986 and 445 amino acids, respectively, and are 93 and 97% similar, respectively, to those of *Salmonella* phage BP63, another broad host range phage that is a component of SalmoPro^®^ (Phagelux, Inc.), a GRAS-certified antimicrobial processing aid for controlling *Salmonella* on foods [61]. A variety of tail-associated accessory proteins were also clustered together at this locus, including tail-fiber assembly proteins, ORFs 46 and 47 (Table S3). This locus also encoded for tail-associated protein products located on the same strand of the genome, indicating they are likely transcribed together as a module [62]. 

SE13 also possesses a variety of DNA metabolism-related genes (e.g., thymidylate synthase, deoxycytidylate deaminase, guanylate kinase, and nicotinate phosphoribosyltransferase) (Table S3). Due to their disparate positions in the genome, it suggests that these genes were acquired by separate horizontal gene transfer events with hosts, prophages or other lytic phages during co-infection, particularly since sewage, from which SE13 was isolated, is an environment facilitating a high frequency of horizontal gene transfer and rearrangements [63].

### 4.6. Genome size, G+C Content and Identification of DNA Metabolism-Related Genes

The cluster analysis revealed that genes encoding for self-replication-related functions were common within and among clusters, and that the number of these elements were associated with the phage genome size. We also observed duplicate copies of genes encoding for a variety of replication-related functions in Clusters 3, 7, 11, and 12, which also comprised phages with larger genomes. Duplicate copies may enhance synthesis of proteins involved in replication, and hence increase phage production and evolutionary fitness.

Although the role of these replication modules in large phages remains unclear, it is possible that larger genomes carry accessory genes that are not essential, but which enable more efficient phage replication. Efficient replication may lead to an increased burst size and/or reduced latent period, both of which are desirable when selecting phages for biocontrol purposes [64]. The fact that Clusters 3, 7, 11, and 12 have a substantially different G+C content than that of their hosts suggests that having more genes for self-replication may be particularly advantageous [20,65,66]. For instance, *Salmonella* has a G+C content of 50% to 52% [20]; whereas, the G+C content of phages in Cluster 3 is ~44%, suggesting the eleven DNA replication elements in these phages may be advantageous (Figure 4). Clusters 7, 11, and 12 also have G+C contents ranging from 39.2% to 44.7%. Moreover, some clusters with a G+C content similar to that of *Salmonella* (e.g., Clusters 4, 5, 6, 8, 10) have fewer self-replication elements (i.e., ranging from zero to three). Concordantly, *Salmonella* phages isolated from dairy farms in rural New York State with G+C contents differing from that of their hosts also harbored anywhere from 1–12 DNA replication elements [20]. 

### 4.7. Identification of Genes Encoding for Putative Virulence Factors

The selection of phages devoid of genetic elements that could pose a risk to human health is critical to biological control applications [9]. Phages can transfer DNA between hosts via transduction [65], which may result in the insertion or deletion of cryptic and/or functional genetic elements, and alter host phenotype [67]. These genetic elements may reside in the phage genome for extended durations until a susceptible host is encountered [68].

Some phages harbored one or more copies of a polymyxin resistance protein ArnC (Table 2), which has also been identified in P22-like viruses 103203_sal5, 146851_sal4, 103203_sal4 and 101962B_sal5, albeit shorter by an amino acid [69]. Naturally synthesized by the bacterium *Bacillus polymyxa*, the polymyxins are a family of last-resort oligopeptide antibiotics used in human medicine that bind to the lipopolysaccharide (LPS) of Gram-negative bacteria, increasing membrane permeability and leakage of intracellular material [70]. Alterations in the moieties comprising the LPS may confer resistance to polymyxins. For instance, the synthesis and transfer of 4-amino-L-arabinose to the LPS is carried out by multiple genes in the *arn* operon [70,71], therefore it is unclear if alterations in one gene in this locus would confer resistance to polymyxin. Phage genomes possessing *arnC* occurred in Clusters 1 and 8, which also comprise putatively temperate phages, suggesting a specialized transduction mechanism (Figure 2). Further, most phages possessing *arnC* were sourced from sewage (SE21, SE22, SE16, SE10, and SE1), a known reservoir of antibiotic resistance genes, and “hotspots” of horizontal gene transfer [64]. Gene *arnC* has also been identified in *Salmonella* phages 22 and 34 isolated in India [72]. However, the absence of antibiotic resistance elements in putatively lytic phages highlights their relatively low frequency of generalized transduction, and suitability for biocontrol. 

We also identified virulence factors in a small subset of phages (Table 2). Virulence factors are naturally found in a broad variety of foodborne pathogens and contribute to enhanced host invasion and environmental fitness [15]. Cluster 3, comprising phages SS3 and SS9 from sediment, carried a gene encoding an identical large virulence protein VriC of 1,613 amino acids (Table 2). SE14, which could not be assigned to a specific cluster, also possessed *VriC* which possessed 99% amino acid identity to that of the Cluster 3 phages. Homologs of VriC have been found to occur elsewhere, for instance, in *Salmonella* phages SFP10 (99.32% amino acid identity), Sh19 (99.01% amino acid identity) [73], and *Escherichia coli* phage PhaxI (99.13% amino acid identity) [74]. Interestingly, *Salmonella* phage 38 appears to possess a truncated form of VriC of 465 amino acids [72]. Although phage-encoded, the origin and function of this protein is unclear, therefore it is unknown if homologs possessing near-identical amino acid sequences would possess the same function. Of the newly isolated phages, SS3, SS9 and SE14 are not classified as temperate, suggesting a generalized transduction mechanism. Although the frequency of generalized transduction is quite rare [67], it has been shown to transfer large genome cassettes and pathogenicity islands [68]. However, it is unclear if these phages possess a high-transducing frequency, and if *vriC* was transduced into a bacterial host would result in a functional virulence factor. 

An exopolysaccharide production protein ExoZ was defined in phages in Clusters 2 and 9. *ExoZ* has been identified in a limited set of phages, including PhWands-1 and PhWands-2 [75]. Functionally, this locus encodes virulent effector proteins, as found in clinically-relevant strains of *Pseudomonas aeruginosa* found in cystic fibrosis patients [66]. Additionally, some species of *Rhizobium* encode a homolog of ExoZ involved in the acetyl modification of succinoglycan, an exopolymer [76]. However, other genes in the *exo* locus are involved in the production of exopolysaccharide [77]; therefore, if introduced alone it is unlikely to cause phenotypic conversion. Nonetheless, as exopolysaccharide production is involved in biofilm formation [77], phages encoding these genes should not be used to control bacterial pathogens, particularly since the rate of transduction is not known.

## 5. Conclusions

Here we described and compared 45 newly isolated *Salmonella* phage isolates on both their basis for biocontrol and biodiversity. Overall, patterns of diversity of the *Salmonella* phages isolated from British Columbia, Canada are complex, although some similarities in the whole genome, MCP sequences and morphotypes occurred among phages isolated from different sites. A novel broad host range phage (SE13) that is genetically distinct from other phage, and which shows no evidence of virulence-associated genes, represents a promising biocontrol agent against *Salmonella*.

We found several putative virulence genes (e.g., *arnC, vriC, exoZ*) in our phages which have only been reported in a few studies [20,72,75]. We also saw evidence for a novel association between genome size and DNA metabolism and GC content in our phages, suggesting links between these genes and enhanced phage replication. The carriage of these genetic elements provides insight into the phage–host interactions and, provided they are appropriately assessed on a genetic level, suggests that our phages may be good candidates for future pathogen mitigation strategies. 

The advent of high-throughput sequencing has led to an explosion of insight into microbial genomes; although, sequencing of phages has lagged behind that of bacteria, despite their critical roles in bacterial evolution. The characterization of this collection of phages contribute to the limited knowledge surrounding phage diversity and phage–host interactions, and will aid with the development of biocontrol strategies against *Salmonella*.

## Figures and Tables

**Table 1:** Closest related sequenced phages to newly isolated phages. The closest related phages (possessing highest E-value and >50% query coverage) and their respective genera were determined through nucleotide homology using NCBI BLASTn.

Newly Isolated Phage	Cluster	Closest Related Phage (NCBI Best Match)	Genus
SE21	1	*Salmonella* phage 103203_sal5	*Lederbergvirus*
SE22	1	*Salmonella* phage 103203_sal5	*Lederbergvirus*
SI23	1	*Salmonella* phage 103203_sal5	*Lederbergvirus*
SF10	2	*Salmonella* phage ST160	*Lederbergvirus*
SI5	2	*Salmonella* phage ST160	*Lederbergvirus*
SI3	2	*Salmonella* phage ST160	*Lederbergvirus*
SS3	3	*Salmonella* phage GG32	*Cvivirinae*
SS9	3	*Salmonella* phage GG32	*Cvivirinae*
SS5	4	*Salmonella* phage vB_SenS_SB3	*Guernseyvirinae*
SS1	4	*Salmonella* phage vB_SenS_SB3	*Guernseyvirinae*
SS7	4	*Salmonella* phage vB_SenS_SB3	*Guernseyvirinae*
SS6	4	*Salmonella* phage vB_SenS_SB3	*Guernseyvirinae*
SF2	4	*Salmonella* phage vB_SenS_SB3	*Guernseyvirinae*
SS8	4	*Salmonella* phage ST3	*Guernseyvirinae*
SI22	5	*Salmonella* phage FSL SP-004	*Peduovirinae*
SW9	5	*Salmonella* phage FSL SP-004	*Peduovirinae*
SI7	6	N/A	N/A
SW5	6	N/A	N/A
SW3	6	N/A	N/A
SE7	7	*Salmonella* phage S147	*Tequintavirus*
SE20	7	*Salmonella* phage Seabear	*Tequintavirus*
SE24	7	*Salmonella* phage S126	*Tequintavirus*
SF3	8	*Salmonella* phage 103203_sal5	*Lederbergvirus*
SF11	8	*Salmonella* phage 103203_sal5	*Lederbergvirus*
SE16	8	*Salmonella* phage 103203_sal5	*Lederbergvirus*
SE1	8	*Salmonella* phage 103203_sal5	*Lederbergvirus*
SE10	8	*Salmonella* phage 103203_sal5	*Lederbergvirus*
SI8	9	*Salmonella* phage ST160	*Lederbergvirus*
SF6	9	*Salmonella* phage SE1	*Lederbergvirus*
SE14	Singleton	*Salmonella* phage S115	*Cvivirinae*
SI1	10	*Salmonella* phage vB_SenS_SB3	*Guernseyvirinae*
SF4	10	*Salmonella* phage vB_SenS_SB3	*Guernseyvirinae*
SF5	10	*Salmonella* phage vB_SenS_SB3	*Guernseyvirinae*
SS10	10	*Salmonella* phage vB_SenS_SB3	*Guernseyvirinae*
SI2	10	*Salmonella* phage vB_SenS_SB3	*Guernseyvirinae*
SS4	10	*Salmonella* phage vB_SenS_SB3	*Guernseyvirinae*
SF1	10	*Salmonella* phage vB_SenS_SB3	*Guernseyvirinae*
SE4	Singleton	*Salmonella* phage ZCSE2	N/A
SE5	Singleton	*Erwinia* phage phiEa21-4	*Ounavirinae*
SE13	Singleton	*Salmonella* phage BP63	N/A
SE11	11	*Salmonella* phage SP01	*Tequintavirus*
SE8	11	*Salmonella* phage SP01	*Tequintavirus*
SE19	12	*Salmonella* phage SP01	*Tequintavirus*
SE18	12	*Salmonella* phage BSP22A	*Tequintavirus*
SE3	12	*Salmonella* phage S147	*Tequintavirus*

**Table 2:** Genotypes and taxonomic assignments predicted from in silico analysis of 45 *Salmonella* phage genomes. Asterisks indicate morphotypes which have been confirmed by transmission electron microscopy [21]. Sources of phages are denoted as follows: sediment (S), cattle feces (F), sewage effluent (E), irrigation water (I), and water tanks from an aquaculture facility (W).

Morphotype Classification				tRNA Genes										*arnC*			*vriC*			*exoZ*		
*Myoviridae*	*Siphoviridae*	*Podoviridae*	Unclassified	Phage	Number	Phage	Number	Phage	Number	Phage	Number	Phage	Number	Phage	Cluster	Source	Phage	Cluster	Source	Phage	Cluster	Source
SE4	SE3	SE1	SI7	SE1	0	SF1	0	SI1	0	SS1	0	SW3	0	SE21	1	E	SS3	3	S	SI3	2	I
SE5	SE7	SE10	SW3	SE3	29	SF2	0	SI2	0	SS3	4	SW5	0	SE22	1	I	SS9	3	S	SI5	2	I
SE13	SE8	SE16	SW5	SE4	0	SF3	0	SI3	1	SS4	0	SW9	0	SI23	1	E	SE14	Singleton	E	SF10	2	F
SE14	SE11	SE21		SE5	27	SF4	0	SI5	1	SS5	0			SE16	10	E				SI8	11	I
SI22	SE18	SE22		SE7	29	SF5	0	SI7	0	SS6	0			SF11	10	F				SF6	11	F
SS3	SE19	SF3		SE8	22	SF6	1	SI8	1	SS7	0			SE10	10	E						
SS9	SE20	SF6		SE10	0	SF10	1	SI22	0	SS8	0			SF3	10	F						
SW9	SE24	SF10		SE11	22	SF11	0	SI23	0	SS9	4			SE1	10	E						
	SF1*	SF11		SE13	0					SS10	0											
	SF2	SI3		SE14	4																	
	SF4	SI5		SE16	0																	
	SF5	SI8		SE18	28																	
	SI1*	SI23		SE19	29																	
	SI2			SE20	29																	
	SS1*			SE21	0																	
	SS4*			SE22	0																	
	SS5			SE24	29																	
	SS6																					
	SS7																					
	SS8																					
	SS10																					

## Supplementary Materials

The following are available online at https://www.mdpi.com/1999-4915/11/9/854/s1, Figure S1: Dendrogram of the major capsid protein nucleotide alignment, Table S1: *Salmonella* strains used for phage isolation and host range determination, Table S2: Sequencing coverage of newly isolated phages, Table S3: Annotated ORFs of the sequenced phages, Table S4: Accession numbers of newly isolated phages, Table S5: Source, indicator strain and plaque morphologies of sequenced phages.
